# Accelerating Well-being for Adolescents Through Transformative Public Policy: A Framework for Action

**DOI:** 10.1016/j.jadohealth.2024.03.013

**Published:** 2024-10

**Authors:** Sarah Baird, Saini Das, Sara Luckenbill, Erin Oakley, Prerna Banati

**Affiliations:** a Department of Global Health, Milken Institute School of Public Health, George Washington University, Washington, D.C.; b Department of Maternal, Newborn, Child and Adolescent Health and Aging, World Health Organization, Geneva, Switzerland

**Keywords:** Adolescents, Causality, Comparative case study, Law, Policy, Regulation

## Abstract

**Purpose:**

As governments around the world are shaping policy responses to advance adolescent well-being and protect their rights, the tools and resources to strengthen policy foundations, and ultimately improve their effectiveness, remain limited. This paper proposes a framework to support policy action with an explicit adolescent focus and applies it to two illustrative case studies to unpack the underlying policy conditions for success.

**Methods:**

We develop an analytic framework with an adolescent lens that focuses on the full policy life-course, from development, to implementation, to evaluation. We then choose two illustrative case studies to apply this framework — 1) abolition of secondary school fees policy in Kenya and 2) age of marriage law in Mexico. These cases were chosen based on the existence of rigorous causal evidence of effect, alignment of salience with expert opinions, broad-based implications for adolescents across contexts, and varied levels of success at achieving intended outcomes.

**Results:**

Our framework identified six key components as critical foundations for adolescent-focused policies: (1) policy features and costs, (2) implementation considerations, (3) participatory approach, (4) inclusion and coverage, (5) policy appropriateness, and (6) monitoring and evaluation, each with key adolescent-specific elements. We find that the majority of the essential policy elements are addressed in the school fees abolition policy (Kenya), but are sparser in the age of marriage law (Mexico). The results also highlight the lack of decentralized monitoring as well as meaningful adolescent engagement at any level of policy development as potential drivers of ineffectiveness of adolescent-centric policies.

**Discussion:**

Our adolescent policy analysis framework can serve as an important tool to define principles in the development of effective adolescent policies. It also can serve as a useful evaluation tool to unpack the ‘black box’ of policy effectiveness when combined with robustly estimated effects.

**Implications and Contribution**

This paper proposes a framework to support policy action for adolescents that links the ‘black box’ of policy development and implementation to the causal literature on effectiveness. This framework can inform policy development processes prospectively, serve as an important evaluation tool for researchers, and guide effective policy development for adolescents.

Adolescent health and well-being are key foundations for economic and social development, yet sectoral action is not always aligned to meet development goals [1]. Societies that invest in healthy and thriving adolescents have high returns on investment [2], strong adult health outcomes [3], low infant and child mortality [4], and improved long term economic development [5]. An important factor determining the well-being of an adolescent is the policy environment in which they live. Policies, laws and regulations can be formulated to protect adolescents from risks (such as laws to prevent early marriage) or empower them by providing opportunities (such as reduction of school fees) [6]. Alternatively, policies and laws can place substantial restrictions on adolescents (e.g., restricting access to sexual and reproductive health (SRH) services for adolescents) [7], constraining their ability to thrive, with potential for impacts across the life-course.

While global strategies such as the 2030 Agenda for Sustainable Development have committed world leaders to deliver transformative public policy change to improve adolescent well-being, policymakers today see many challenges to achieving such results. Not only is substantial funding often required over a long timeframe, but effective impact often requires coordinated action across structures (e.g., infrastructure, human resources) and sectors (health, education). Moreover, it is likely that investments in one area affect investments and behavior in others [8]. The fact that a long lag time can exist between policy enactment and results can both limit political will to implement the appropriate policy and complicate evaluations of effectiveness.

Despite these challenges, policy interventions during adolescence have the potential to be successful due to rapidly changing brain development that, combined with interaction with the social environment, shapes outcomes during this life stage and beyond [3,9]. This recognition of the potential ‘triple dividend’ of intervention during adolescence, which can have impacts on the adolescents of today, into their future as they become adults, and for their children - has led to increased financial investment in and policy attention on adolescence, and increased attention to rigorous research in this space [3].

While the evidence base on effective interventions for adolescents has increased considerably over the past decade [10] and has led to some important programmatic conclusions (see Bergstrom and Özler [11], Malhotra and Elknaib [12], and García & Saavedra [13] for reviews and meta-analysis), there is still limited evidence on what will work sustainably at scale. The lack of capacity to scale even the most successful adolescent interventions, such as human papillomavirus vaccination [14], creates substantial challenges in progress towards the sustainable development goals and beyond.

While interventions are an important part of improving adolescent well-being globally, arguably national policies and laws are likely to be more influential drivers of sustained transformative change [15]. This includes policies, laws and regulations that affect adolescents directly (e.g., universal secondary education) and indirectly (e.g., through social protection to the household). While we know that laws and policies have the potential to play a huge role in the trajectory of an adolescent, the ability to draw broad policy conclusions from the evidence base remains somewhat limited. This is due to three main factors: (i) many evaluations of laws and policies do not disaggregate by age so it is unclear if impacts accrue to adolescents, (ii) rigorous evaluations of laws and policies are often either not feasible or require more complicated quasi-experimental methods, and (iii) and even when a rigorous study does exist, it is often from a single policy change in a single country (e.g. introduction of minimum age of marriage in Mexico [16] or abolition of secondary school fees in Kenya [17]). [18,19] For example, when apparently similar policies are implemented in separate locations with divergent results (for instance, Universal Primary Education policies show differing impacts on adolescent girls' outcomes related to child marriage [20] and experiences of sexual violence in Uganda compared to Malawi [21]), it can be difficult to ascertain which elements of the policy or context underlie success. This is further exacerbated by the fact that the peer reviewed literature often does not contain the necessary information to unpack the ‘why and how’ of policy effectiveness, limiting translation of rigorous research findings into concrete future policy recommendations.

This paper builds on multidisciplinary scholarship [[22], [23], [24], [25], [26], [27], [28], [29], [30]] in the emerging field of adolescent health policies and systems research and aims to contribute to the evidence lacuna by proposing a framework for policy analysis with an explicit adolescent lens. The hope is that this framework can be used to make visible the contents of the ‘black box’ of policy effectiveness to guide future adolescent policy development and provide a tool for future policy development. We apply this policy framework to two illustrative cases where there is rigorous evidence for causal impacts and causal estimation. We use a rigorous realist approach [31], which strengthens conventional reviews by examining empirical findings in addition to narrative methodologies of ‘context-mechanism-outcome’ configurations. This approach assists in identifying alignment between implementation mechanisms and overall effectiveness and presents a rigorous means of applying the proposed framework.

The cases were identified as part of a systematic scoping review [32] that identified the evidence base on the causal impact of national or subnational laws and policies on adolescent well-being globally, and then triangulated these findings with expert opinions (we discuss both of these in more detail after describing the framework). Using these cases we unpack the components of policies, laws and regulations that have been successful (or not) to improve the lives of adolescents globally. We do not suggest that implementation of framework components should be sequentially introduced, nor do they present a straight line to impact. Rather, we seek to provide guidance to policy makers on the underlying policy conditions necessary (but not sufficient) for success.

### Framework for comparative case study analysis

We first develop a framework with an adolescent lens to guide this analysis. We reviewed the literature for existing well-established health policy analysis frameworks [[33], [34], [35], [36], [37], [38]]. We also consulted with Youth Commissioners from the Second Lancet Commission on Adolescent Health and Wellbeing [39], who provided an important youth lens to the framework development.

Many of these frameworks were identified at an expert technical consultation hosted by the World Health Organization/HRP to review 10 years of progress on SRH in young adolescents in developing countries held in Geneva in August 2023. The following frameworks were consulted and included. Shiffman [33,34] in his foundational work on health policy analysis describes the role of actors, framing, political context and ‘issue characteristics’ as central to the policy evaluation model. The work of the Center for Global Development (CGD) [35] focuses on components of policy sustainability, the need for visionary leadership, policy appropriateness as well as strong implementation management. The CGD [35] model also raised the important question on how to address resistance in the face of policy innovation, with relevance to SRH policy. World Health Organization and ExpandNet [36] built on these further to include the crucial role of policy advocacy, the appropriate use of evidence, data and information in policy design and execution, robust organizational processes, adequate and sustained mobilization of resources, and the importance of monitoring and evaluation. Hadley et al. [37] stresses the value of iterating and tailoring strategy to reflect implementation updates. We found a number of additional references pointing to the need for an iterative design between policy and implementation to support policy learning initiatives [40] and recognizing the importance of multisectoral policy intervention including by recommending the embedding of policy strategy in wider government programs [1,41,42]. Finally, we draw on the Jacobs and George [38] framework which deepened social and rights-based elements into their policy evaluation model, adding the lens of vulnerability, addressing symptoms or transforming underlying drivers and addressing the degree to which the policy is inclusive of diversity. Many of the frameworks shared common features (such as strong leadership and effective implementation), though lacked specificity for adolescents. Drawing together the learnings from these policy analysis frameworks, we identified the key features relevant to policy analysis broadly, adapted these features for adolescent social development, and added features we identified as important from youth leaders.

The resulting framework seen in Figure 1 is organized around six headings: (1) policy features and costs, (2) implementation considerations, (3) participatory approach, (4) inclusion and coverage, (5) policy appropriateness, and (6) monitoring and evaluation. Figure 1 describes the key dimensions of our proposed analytic framework. While many more elements could have been included, it was considered important to prioritize key analytic features where actions could be taken to strengthen policy making processes.

**Figure 1:**
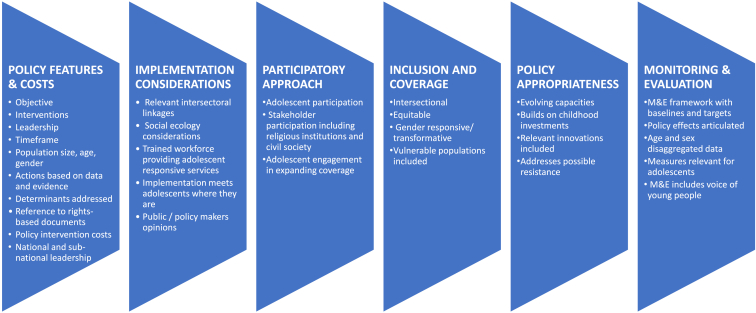
A proposed policy analysis framework for adolescents (This graphic draws from the policy analysis frameworks of Shiffman [20,21], Center for Global Development [22], WHO and ExpandNet [23], Hadley et al. [24], and Jacobs and George [25]).

## Results: Policy Cases

We now apply this framework to two illustrative cases. As mentioned above, we selected cases that were identified in a systematic scoping review (described in detail in Baird et al. [32]) focusing on rigorous causal evidence of the impact of exposure to policies and laws during adolescence. Specifically, the review set out to answer the question “What is the evidence base on the impact of exposure to national or subnational laws and policies during adolescence on adolescent (or later life) well-being globally?” Focusing on studies in English published between 2010 and 2023, we searched a set of multidisciplinary databases using keywords adapted to the search engine. We chose not to delineate specific outcomes or policies as long as the policy or law took place at a national or subnational level and the exposure took place during ages 10-19. Our main inclusion criteria are that the studies include methods that create a valid counterfactual, and thus could reasonably capture causal impacts. By not restricting on type of policy or outcome domain, but focusing on any national and subnational policies that target adolescents, this search provides a comprehensive assessment of the evidence base for policy exposure during adolescence. For full details on the registered protocol see Baird et al. [32].

To triangulate the systematic review, we also sent emails to over 100 experts in the adolescent field to solicit examples of exemplars, positive or negative (see Table 1 for a summary of the affiliation of the 32 experts that responded).

**Table 1:** Organizational affiliation of expert consultant respondents

Academia	Johns Hopkins University (1)	Tulane University (1)	Karolinska Institutet (1)	Harvard University (1)
	George Washington University (3)	Tufts University (1)	University of Auckland (2)	University of Washington (2)
Nonprofit and think – Tanks	Center for Global Development (4)	Save the Children (1)	Population Council (2)	
Philanthropic foundations	Bill & Melinda Gates Foundation (2)	Gates Ventures (1)		
United Nations agencies	World Health Organization (1)	UNESCO (1)		
Research institutes	Overseas Development Institute (1)	African Population and Health Research Center (1)	Murdoch Children's Research Institute (1)	Burnet Institute for Medical Research (1)
Youth leaders	Second Lancet Commission on Adolescent Health and Wellbeing Youth Commissioners (4)			

These are case studies where the experts believe there is evidence that a given policy or law either transformed the lives of adolescents (‘exemplar’), had no effect (‘neutral’), or in fact had an adverse effect (‘anti-exemplar’). We compiled a database of all responses and cross-checked with findings from the systematic scoping review evidence. Interestingly, for the majority of examples identified by the experts, while documentation existed in many cases, we were not able to find any convincing causal empirical quantitative evidence of effectiveness.

From this cross-referenced list, two cases were selected with rigorous causal evidence of impact based on two main criteria. First, we focused on high impact policies with potential widespread application applied in geographically diverse settings. We selected policies that have implications for adolescents across contexts and selected cases that operate in different contextual settings. The first case in Kenya is an example of fee subsidies for secondary school in a sub-Saharan African context [43]. Such a policy intervention has been seen in a number of African and Asian countries. The second from Mexico is a case of prohibition of early marriage [44]. Again, this type of policy has been applied in many countries across Latin America, Africa and Asia to date. Second, we were interested in comparing different levels of success at achieving intended outcomes. For this, we selected cases that have been successful at achieving the intended outcome (Kenya [43]) as well as a mixed success example (Mexico [44]).

In the Kenya example, Muchiri [43] evaluated first births for women ages 15-18 from two cohorts, adolescents born in 1990–1993 who were directly impacted by the abolition of secondary school fee policy, and those who were born earlier 1985-1988, who reached adolescence when the policy was not in place. Both cohorts were surveyed in 2014, when the treated (policy-exposed) group was 21–24 years old, and the control group was aged 26–29 (13 years post policy). Exploiting the timing of the reform, Muchiri [43] demonstrates the role of the policy on reducing teenage motherhood, estimating that this decreased by about 5 percentage points after the policy's implementation. The paper also describes the positive potential long-term economic growth as well as impacts on poverty reduction, and reduction of social welfare dependency [43]. The results from the Mexican example are more mixed [44]. Analysis shows that birth rates among married women under the age of 18 did fall 9 years after the application of the law prohibiting marriage under 18. However, consensual unions continued to thrive in Mexico and wider Latin America, due to growing acceptance towards this practice [44]. Analysis suggests that in places where cohabitation is socially acceptable, the minimum age of marriage law is likely to be ineffective as marriages are displaced by early unions which may also bear negative consequences [16]. Lyn and Rainer [44], using a staggered difference in difference approach on data in Mexico, demonstrate that the law had no overall effect on total teenage birth rates and girl's school attendance.

With these two illustrative cases – one policy and one law – we then use our framework (Figure 1) to describe the underlying conditions that have contributed to the success (or lack thereof) of the policy or law on adolescent outcomes. Since this information is often not found in the peer reviewed literature, we draw largely from publicly available grey literature. In addition to Google searches, this involved exploring digital resources made available by government authorities (such as original policy documents or law edicts), online policy repositories or global policy think tank publications, including at the WORLD Policy Analysis Center (https://www.worldpolicycenter.org/), the Center for Global Development (CGD) (https://www.cgdev.org/), World Bank Policy Research reports (https://www.worldbank.org/en/research/brief/policy-research-reports) and relevant United Nations (UN) agencies. While the searches were not systematically conducted, they sought to gather information relevant to the cases as comprehensively as possible. As with most grey literature, the information gathered was largely not peer reviewed and of variable quality. Searches were conducted in English and Spanish. Due to limitations within the research team, we were unable to search in Swahili but do not anticipate that this would significantly impact our findings as English is one of two national languages, and widely used in Kenya.

Table 2 systematically analyzes each case applying them to the six key components of our policy framework. We used a rigorous realist approach [31], which strengthens conventional reviews by examining empirical findings in addition to narrative methodologies of ‘context-mechanism-outcome’ configurations. Syntheses aimed to identify alignment with the framework's components by synthesizing policy descriptions, evaluation data and process management information. Our synthesis sought to understand how features of policies, resources, scale up and related intervention elements influence policy action and could ultimately influence policy success. We then analyzed how well evidence from the quantitative evaluations, including overall effects, mediators, moderators and necessary conditions for effectiveness were aligned with the narrative summary. Where evidence allowed, we drew lines of argument from multiple studies in order to build a bigger picture of mechanisms of action, and where discordant information was uncovered, we presented these as inconclusive.

**Table 2:** Analysis of Kenya and Mexico cases

	Kenya	Mexico
	Subsidized secondary school policy 2008	Ley general de los derechos de niñas, niños y adolescentes 2014
Policy features and costs		
What is the policy/legal objective?	To increase access to secondary education and contribute to Kenya's sustainable development by the provision of subsidized, high quality secondary education [45].	To affirm the right of all children adolescents below the age of 18 years to live in conditions that allow their development, well-being and healthy and harmonious growth, both physical and mental, material, spiritual, ethical, cultural and social by raising the minimum marriageable age to 18 years without exceptions [48].
What are the proposed interventions?	The 2008 Secondary Education policy was introduced in Kenya to expand secondary education by reducing fees and increasing education capacity. The fee reductions were introduced for grades 9-12 and contributed about 40% of the household cost of sending a child to secondary school. Policies to increase the capacity of public secondary schools, increase the size and number of classes per grade and also construct new secondary schools were also implemented [17,45].	The law establishes 18 as the minimum age of marriage and was amended in 2019 to remove all exceptions to the marriage age [48].
Who leads the policy/law?	Kenya Ministry of Education	Executive Secretariat of the National System for the Comprehensive Protection of Children and Adolescents (SIPINNA)
What is the timeframe?	2008- present	2014, amended in 2019
Are the proposed policy actions based on evidence and data?	There has been a situation analysis of secondary schooling undertaken in support of the policy [45].	Information on the situation of children was prepared for the Committee on the Rights of the Child in 2015 [49] and submitted as part of the periodic review. The information provided related to teen pregnancy rates, rates of violence among children, as well as secondary school completion rates. In order to support the campaign, UN Women analyzed nationally produced information, based on the 2014 National Survey of Demographic Dynamics (ENADID) of the National Institute of Statistics and Geography (INEGI) [50].
Are determinants of adolescent health and well-being addressed?	Only family poverty is considered a determinant in the strategy of fee reduction [45].	The Convention on the Rights of the Child concluding comments notes the need to address child marriage including forced marriage and disproportionate impacts for girls belonging to indigenous communities [49].
Does it reference rights-based documents (e.g. CRC)?	Yes. CRC, CEDAW, Convention on the elimination of the worst forms of child labour and other key human rights documents are referenced [45].	Not explicitly, though UN documentation notes the Convention on the Rights of the Child Concluding comments [49] motivated the change in law.
Are the policy interventions costed?	Not in the policy documents.	Not in the legal documents.
Implementation considerations		
Does the policy address relevant intersectoral linkages across domains of adolescent well-being?	Yes. Early marriage is acknowledged as a challenge to secondary education. The way forward notes the need for a school reentry policy for girls who drop our early due to early pregnancy or marriage. Child labor is acknowledged as a challenge to inclusive education. The concept of inclusive education is defined in the policy, with clear description of children in especially difficult circumstances [45].	Not explicitly. However, the campaign for legislative change included the challenge of generating comprehensive public policies for education and social protection that address the structural causes of inequality, poverty and discrimination towards girls [50].
Are all relevant parts of the social ecology of adolescents addressed (parents/schools/community etc.)?	Yes. The policy states the aim of developing partnerships with parents, communities, civil societies, the private sector and other stakeholders to ensure effectiveness. Parent's financial limitations are also taken into account [45].	The recommendation from the Convention on the Rights of the Child notes that Mexico should undertake comprehensive awareness-raising programs on the negative consequences of child marriage on girls, targeting in particular parents, teachers and indigenous leaders [49].
Does it include workforce trained in adolescent responsive service provision, including privacy and confidentiality where needed?	Partial. Teacher training involves a modernized curriculum, including a child centered approach, and introduction of HIV/AIDS education, and education on drug and substance abuse [45].	No evidence.
Does implementation meet adolescents where they are (location, timing)?	Yes. Policy involves the expansion of access through building of more day secondary schools [45].	No evidence.
Participatory approach		
Are adolescents participating in the policy process?	No evidence.	No evidence.
Are relevant stakeholders such as parents and teachers participating in the policy process?	No evidence.	A joint strategy was prepared which included 13 civil society organizations and 12 United Nations agencies, as well as the Presidency of the Republic, the National Conference of Governors and the Mexican Congress [50].
Are young people engaged in expanding coverage?	No evidence.	No evidence.
Inclusion and coverage		
Does it acknowledge intersections (gender, age, other)?	Yes. Gender is identified in the conceptualization of inclusive education. Gender parity, and creation of a gender responsive learning environment are identified as key objectives, including the retention of girls in school. Sex and regional disaggregated data is presented. Reference to disability throughout the document [45].	Yes. The situation in indigenous and poor communities was a driving force for change [50].
Does it strive for equity (e.g. for girls and boys)?	Yes. Equal access to education is a stated outcome.	The law applies evenly to boys and girls, harmonizing a previously unequal law where minimum marriage ages were 14 years for girls and 16 for boys [50].
Does the policy acknowledge gender/strive to be responsive/transformative?	Yes. Reference to gender responsiveness throughout the policy [45].	Yes. The situation of gender equality is motivated in the Convention on the Rights of the Child review [50].
Does it explicitly mention vulnerable populations (e.g. ethnic minorities, refugee populations)?	Yes. Reference to immigrant and refugee populations but no explicit strategies identified [45].	Yes. Indigenous populations are explicitly mentioned [48].
Policy appropriateness		
Does the content acknowledge evolving capacities?	No. No explicit mention of evolving capacities.	No evidence.
Does it build on earlier investments in childhood?	Yes. The policy takes an age and life course approach, acknowledging earlier investments in child education as important to build upon [45].	No evidence.
Are innovations relevant for young people considered (e.g. digital)?	Yes. Aim involves integration of alternative models of providing education including the use of ICT so as to build a foundation for technological and industrial development; digitalization of curriculum to enhance e-teaching and e-learning [45].	No evidence.
Is there effort to address resistance and how was this managed?	No evidence.	No evidence.
Monitoring and evaluation		
Is there a monitoring and evaluation framework with baselines and targets identified?	Policy document notes alignment to Global Development Goals [45].	Not in the legal documents.
What are the effects of the policy?	The policy increased female educational achievement, delayed childbirth and related demographic behaviors, and shifted employment away from agriculture towards skilled work [17,46,47].	Reduction in marriage rates under 18, and related consequences such as teenage pregnancy, violence reduction and secondary school completion [44].
Is there age and sex disaggregated data used for monitoring and evaluation?	Yes [45].	Not in the legal documents.
Are indicators of relevance to young people included?	No evidence.	No evidence.
Does M&E include the voice of young people?	No evidence.	No evidence.

The text below provides an overview of the cases, highlighting key features of the framework that may have contributed to success of the policy intervention.

### Case 1: abolition of secondary school fees policy in Kenya and associations with education, demographic and short-term labor market returns

On the heels of noteworthy impacts of the abolition of primary school fees in Africa on school enrollment [51], in the last two decades several countries in sub-Saharan Africa including Ghana, Madagascar, Malawi, Sierra Leone, Togo, Zambia, Kenya, Rwanda, South Africa and most recently South Sudan, have announced free secondary education policies [52]. These countries have introduced free or subsidized secondary education as a strategy for human capital formation. The documented social and economic benefits of secondary education are significant, and evidence suggests causally related to higher earnings and positive health outcomes [53]. Impacts are even greater for girls, where secondary education has been associated with greater agency, empowerment, and lower incidences of early marriage and teenage pregnancy [43,54,55].

The case example from Kenya demonstrates that education reform can have impacts beyond the education sector. The findings show that these reforms not only impact education outcomes, but can also have a positive causal impact on other domains, namely reductions in teenage motherhood and increased labor market participation [17,51,56]. The 2008 Free Day Secondary Education Policy [45] was introduced to expand secondary education for both boys and girls by reducing fees and increasing education capacity. The fee reductions were introduced for grades 9-12, building on free public primary school for grades 1-8 that was introduced 5 years earlier. The reductions were substantial, amounting for about 40% of the household cost of sending a child to secondary school [17]. The government also implemented policies designed to increase the capacity of public secondary schools, increasing the size and number of classes per grade and also constructing new secondary schools. Over 1.9% of the Kenyan national budget was earmarked for the tuition component of the program [45].

The policy shows a strong focus on equity and inclusion, and while the policy applied to both boys and girls, girls' education in particular is singled out as a key policy objective. There is also thoughtful analysis of human resource needs and related costs to address these gaps. This is particularly important in the wake of learnings from universalizing primary school education, where removals of economic barriers to education increased class size in some cases, and ultimately compromised education quality [51]. That said, long term predictable financing will be required to allow schools to replace income from loss of school fees and maintain quality. Challenges to transitioning to university education and technical and vocational training is well considered, with a good analysis of bottlenecks and reference to interoperability across the parts of government responsible for these. Deeper analysis of labor market constraints and supporting transitions to the workplace could be included. Realizing the economic potential of expanding secondary education is likely to require careful planning and wide negotiation in order to ensure expectations of secondary school leavers are realized and labor market returns are maximized. With the exception of adolescent and stakeholder participation, where no documentation was available, Kenya's inclusive education policy satisfies the majority of the components described in Figure 1, as seen in Table 2.

### Case: Mexico age of marriage

Increasingly, countries are adhering to human rights standards to prohibit and close legal loopholes that permit marriage under 18. Girls remain disproportionately affected by early marriage, with one in five young women aged 20–24 years old married before her 18th birthday, compared with one in 30 young men [57]. Evidence from sub-Saharan Africa has suggested that girls married before 18 are more likely to experience teenage pregnancy than those married after 18 [58,59], more likely to experience violence [60], and less likely to complete basic schooling [61], with consequences of slower national economic growth [62]. Arthur et al. [63] reviewed evidence on legal provisions shaping child marriage and found that in 23 of 191 countries, girls are legally permitted to marry under the age of 18. This increases dramatically when parental or guardian consent is provided or in the cases of customary or religious law exceptions. With these cases, 102 out of 191 countries permit marriage for girls under 18.

In 2014, the Federal Congress of Mexico sanctioned a law setting the minimum age for marriage for both women and men at 18 and urged all federal entities to reform their legislation to incorporate this change [48]. Since marriage laws are typically the purview of states, states can ultimately decide whether to adopt the reform or not. Some states had already adopted such laws, while others were slower to do so. By the end of 2015 only eight states had changed their marriage laws to comply with federal legislation. Concern was expressed in the concluding observations by the UN Committee of the Rights of the Child at the periodic reporting for Mexico, held in 2015 [49]: ‘While noting that, in accordance with article 45 of the General Act on the Rights of Children and Adolescents, federal and state laws should set the minimum age for marriage at 18 years for both boys and girls and that the Federal Civil Code has already been modified accordingly, the Committee was concerned about the effective implementation of this provision at the state level. It is also concerned about the high prevalence of child marriage and about reported cases of forced marriage, especially involving girls belonging to indigenous communities.’

The Executive Secretariat of the National System for the Comprehensive Protection of Children and Adolescents in Mexico joined an advocacy effort led by international organizations and civil society organizations to promote the uptake of the law at federal and local levels. This included the Ministry of Health; the National System for the Integral Development of the Family; the Undersecretariat for Human Rights, Migration and Population of the Ministry of the Interior; the National Women's Institute; the National Population Council and the National Council for the Prevention of Discrimination as well as international organizations such as United Nations Children’s Emergency Fund and UN Women [48]. This resulted, in 2019, with the addition of a specific amendment to the law to prevent child marriage *without exceptions*. In this amendment, local and family authorities are no longer able to grant dispensations to marry before the age of 18.

In the Mexico case, policy objectives and timelines were clear, though implementation particularly at subnational levels was not articulated, leaving states able to enforce measures inconsistent with the national general law. While the underlying motivation included the desire to address populations at risk, including girls from indigenous communities, the decentralized nature of the application meant these legal protections were not necessarily available universally for all adolescents at risk of early marriage, though over time these provisions were expanded to all states.

## Discussion and Conclusion

Grounded in foundational health policy frameworks [[33], [34], [35], [36], [37], [38]], this paper develops a policy analysis framework with an explicit adolescent lens. First, this age-based framing helps illuminate factors that are likely critical for adolescent policy effectiveness across the policy lifespan from development to monitoring and evaluation. For example, it highlights the value of strengthening adolescent responsive systems (such as seen in the case of the education system reform in Kenya) through the introduction of well-designed public policy, and for implementation to meet marginalized and vulnerable adolescents (such as those out of school or living with disability), both factors often mentioned in the failure of adolescent interventions or policies to have their desired impact [64]. Similarly, it touches on the importance of a participatory approach to policy making, including multiple line ministries, civil society and relevant national and subnational actors. Adolescent participation that is at the core of the movement towards meaningful youth engagement, is still lacking in the development of most policies targeting adolescents [65]. Inclusion of adolescents within the policy process was identified as a key component in the framework, yet application of the case studies highlighted the absence of this important group in the development of policies that affect them.

Second, the framework draws attention to the fact that goal-oriented policies (specifically the Kenya case in this study), implemented at the grassroots level have the potential to achieve more adolescent-centric outcomes than a centralized policy with limited involvement from local government.

Third is the need for sustainable policy implementation to operate through robust structures across levels and sectors of government. In the case of Kenya, intersectoral engagement was motivated by the informed by the qualitative and quantitative analysis of barriers that operated across domains of adolescent life. Our analysis also pointed to the value of recognizing the roles of duty bearers such as parents and teachers in implementing policies for adolescent well-being.

Lastly, the framework also draws attention to the importance of an age- and gender-disaggregated monitoring and evaluation strategy. Age and sex-disaggregated data were required to evaluate the impacts of the two policies studied in this paper. While this may at first glance appear obvious, it is a common plea throughout the adolescent research and policy community [66,67]. Many policies and laws that impact the lives of adolescents may target broader age ranges and it is crucial we are able to unpack effects for this age group (ideally further disaggregated by young and older adolescents). Otherwise, we rely on average effects that may mask either positive or adverse effects specifically for adolescents. Further, even if the policy is adolescent focused, rigorous evaluation strategies frequently rely on secondary data that does not have the appropriate age disaggregation [68]. This is one of a multitude of factors that limit robust quantitative evidence for the effectiveness of policies and laws. This means that policy advances can be driven by the opinions of insiders or experts, as opposed to evidence. This critique can be extended beyond measurement. Policies and laws that impact the lives of adolescents may target broader age ranges and may not be sufficiently focused on age or gender related specificities. Policies are likely to benefit from recognition of the different social ecologies surrounding, for example, a younger male adolescent or an older female adolescent, and address these in the design and implementation of policies.

Our study has a number of important limitations. Firstly, we are cognizant that the proposed policy conditions presented in the framework, and expounded in the case studies, may not be the only factors that contribute to success. It is entirely plausible that implementation realities and factors such as decentralization, political and international pressures may be important predictors of policy success. More could be gathered on these and other items through in depth interviews, and these would be a nice addition when feasible, but are beyond the scope of timelines and budgets for many trying to unpack the ‘black box’ of implementation. Secondly, our framework ultimately included six key dimensions, each populated with a set of priority elements. Our team prioritized dimensions and elements that we felt were most relevant for adolescent health and well-being, but another research team may have selected other areas to focus on. Third, while we used a set of specific criteria to select our two cases, there were other cases we could have chosen. While we believe the findings from these two cases provide a useful illustration of the application of the policy analysis framework, analysis of all relevant cases would have perhaps allowed for some broader generalizations. This could be fodder for future research. Fourth, we did not systematically search for the grey literature used to conduct the policy analysis for our two cases.

Ultimately, rigorous quantitative analysis of policies and laws combined with robust policy analysis is critical for unpacking the ‘black box’ of the how and why of policy effectiveness. Bringing an age- and gender-lens to this analysis is needed to ensure that we develop policies and laws that are responsive to the needs of adolescents and designed to maximize the likelihood for success, particularly given the importance of the policy environment for adolescent well-being and realizing the ‘triple dividend’. Ultimately, we see this as a framework for action that can be used to accelerate well-being for adolescents through transformative public policy.
